# Effects on pig immunophysiology, PBMC proteome and brain neurotransmitters caused by group mixing stress and human-animal relationship

**DOI:** 10.1371/journal.pone.0176928

**Published:** 2017-05-05

**Authors:** Daniel Valent, Laura Arroyo, Raquel Peña, Kuai Yu, Ricard Carreras, Eva Mainau, Antonio Velarde, Anna Bassols

**Affiliations:** 1 Departament de Bioquímica i Biologia Molecular, Facultat de Veterinària, Universitat Autònoma de Barcelona, Cerdanyola del Vallès, Spain; 2 Servei de Bioquímica Clínica Veterinària, Facultat de Veterinària, Universitat Autònoma de Barcelona, Cerdanyola del Vallès, Spain; 3 IRTA, Subprograma de Benestar Animal, Monells, Spain; 4 Departament de Ciència Animal i dels Aliments, Facultat de Veterinària, Universitat Autònoma de Barcelona, Cerdanyola del Vallès, Spain; University of Edinburgh, UNITED KINGDOM

## Abstract

Peripheral blood mononuclear cells (PBMC) are an interesting sample for searching for biomarkers with proteomic techniques because they are easy to obtain and do not contain highly abundant, potentially masking proteins. Two groups of pigs (n = 56) were subjected to mixing under farm conditions and afterwards subjected to different management treatments: negative handling (NH) and positive handling (PH). Serum and PBMC samples were collected at the beginning of the experiment one week after mixing (t0) and after two months of different handling (t2). Brain areas were collected after slaughter and neurotransmitters quantified by HPLC. Hair cortisol and serum acute phase proteins decreased and serum glutathione peroxidase increased at t2, indicating a lower degree of stress at t2 after adaptation to the farm. Differential gel electrophoresis (DIGE) was applied to study the effects of time and treatment on the PBMC proteome. A total of 54 differentially expressed proteins were identified, which were involved in immune system modulation, cell adhesion and motility, gene expression, splicing and translation, protein degradation and folding, oxidative stress and metabolism. Thirty-seven protein spots were up-regulated at t2 versus t0 whereas 27 were down-regulated. Many of the identified proteins share the characteristic of being potentially up or down-regulated by cortisol, indicating that changes in protein abundance between t0 and t2 are, at least in part, consequence of lower stress upon adaptation to the farm conditions after group mixing. Only slight changes in brain neurotransmitters and PBMC oxidative stress markers were observed. In conclusion, the variation in hair cortisol and serum APPs as well as the careful analysis of the identified proteins indicate that changes in protein composition in PBMC throughout time is mainly due to a decrease in the stress status of the individuals, following accommodation to the farm and the new group.

## Introduction

It is well known that pigs are social and under some circumstances hierarchical animals [1], hence mixing unfamiliar animals on the same pen produce a stressful situation with physiological consequences on the individuals [2–6]. Furthermore, there is considerable research interest in human-animal relationship. In pigs, as well as in other species like dairy cows and poultry, it has been shown that negative human-animal interactions can markedly alter the productivity and the welfare of animals by affecting the animal fear towards humans [7–9]. On the other side, positive interactions such as pats, slaps or talking to the animals reduce fear and human avoidance [9]. For example, the presence of a familiar human, providing gentle handling, may calm the animals in potentially aversive situations (e.g. isolation, tethering, rectal palpation, insemination) thereby reducing distress and the risk of injury to animals and humans [10]. In this work, the effect of human handling after a stressful situation associated to mixing individuals during farm adaptation in pigs is studied.

Despite its importance, information on laboratory biomarkers for the objective evaluation of stress and welfare is scarce. Stress hormones as cortisol have been used, together with other markers such as acute phase proteins (APP) [11–14], but there is a need for more and more specific markers, especially for chronic stress. For the search of new biomarkers, proteomic technologies have become very useful and, recently, these techniques have been used in many studies on farm animals [15–18] and specifically, in stress and welfare-related issues [11,19–22]. Proteomics has the great advantage to look at proteins, which are the real biological actors in the cell and the organism, whereas genomics or transcriptomics analyze genes or RNA transcripts, which can or cannot be translated. Up to now, the majority of studies looking for biomarkers have been performed in serum or plasma, but there are problems associated with the interference of highly abundant proteins in these samples [23,24]. Peripheral blood mononuclear cells (PBMC) are an interesting alternative as a convenient biological sample to monitor processes that lead to subtle physiological changes difficult to detect in plasma samples, especially those related with the involvement of the immune system. Protein expression by PBMCs have been characterized in porcine [25,26] and this sample type has been used to gather information about a series of biological conditions in pigs, for example, pregnancy [27], influence of diet [28,29] or heat stress [30].

On the other side, the noradrenergic, dopaminergic and the serotonergic pathways in the central nervous system (CNS) are the most important and well characterized systems underlying the response to stress, fear and reward, among others. The central nervous system controls the action of endocrine glands through the release of catecholamines, indoleamines and other transmitters which can be excitatory or inhibitory mediators. Amygdala, hippocampus and prefrontal cortex (PFC) are recognized to play a role in the stress response organization. In these structures, stressors produce changes in extracellular concentrations of different neurotransmitters (NTs) leading to activation and modulation of processes to cope with stress. These areas have an indirect output to the hypothalamus, which acts modulating the final stress response through the sympathetic nervous system and the activation of the hypothalamic-pituitary-adrenal (HPA) axis. Therefore, the stress response involves the interaction among theose areas through NTs, especially catecholamines (noradrenaline (NA), dopamine (DA)) and the indoleamine serotonin (5-HT). DA is metabolized to homovanillic acid (HVA) and 3,4-dihydroxyphenyl acetic acid (DOPAC), whereas 5-HT is metabolized to 5-hydroxyindoleacetic acid (5-HIAA)[31–34].

The main goal of the present work was to analyse changes in the PBMC proteome of the pigs throughout the stocking period in the farm after a stressful episode of animal mixing, and to ascertain whether how animals are handled can influence these changes. As a second goal, the influence of human-animal relationship on brain neurotransmitters (noradrenergic, dopaminergic and serotonergic systems) and PBMC oxidative stress markers (superoxide dismutase (SOD), protein carbonylation) was evaluated.

## Materials and methods

### Experimental design

In this study, 56 female pigs ((Landrace x Large White) x Piétrain, free of the halothane gene) coming from 21 sows from the same commercial farm were used. At 9 weeks of age, pigs were transported to the experimental facilities of IRTA (Institut de Recerca i Tecnologia Agroalimentàries, Monells, Spain) and randomly housed in two rooms of four pens with seven pigs in each. The pens (5 x 2.7 m) had fully slatted floors with natural light conditions at a constant environmental temperature of 22 ± 3°C. Each pen was provided with one steel drinker bowl (15 x 16 cm) connected to a nipple and a concrete feeder (58 x 34 cm) with four feeding places. Pigs had water and food *ad libitum*, and pigs were provided in all pens with chains as material for manipulation. Pigs were inspected daily and no health problems were observed during the experimental period. The study was approved by the Institutional Animal Care and Use Committee (IACUC) of IRTA.

**Fig 1:**
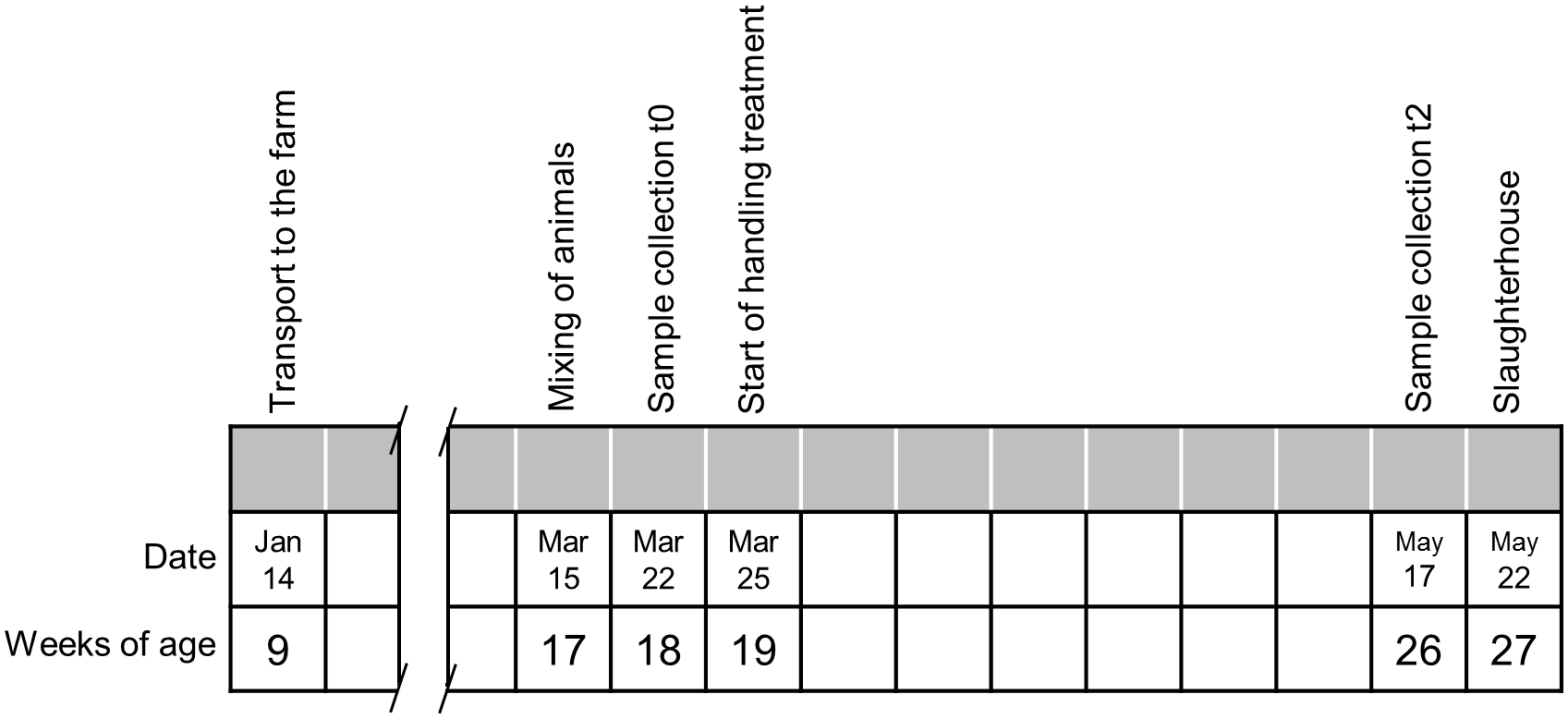
Experimental timeline showing dates for animal handling, treatments and sample collection.

Treatment and sample collection were carried out as shown in Fig 1. Pigs were mixed at week 17 of age and one week later the first samples were collected (t0). Handling treatments started three days later and a second sample was collected after two months (t2). The pigs in one of the rooms received positive handling (PH) and the pigs in the other room received negative handling (NH). During this time, one experimenter entered five days per week for approximately 30 min each day in each room between 9:00 am and 5:00 pm. The time to perform the handling treatment and the order to enter the room and the pens was randomly distributed each day. The PH treatment consisted in entering the room slowly and letting the pigs looking at the experimenter with binocular vision before entering the pens. After 1 min, the experimenter entered the first pen and walked slowly around its perimeter. Then, the experimenter stopped at one corner of the pen and adopted a steady squat posture for 5 min, touching and interacting gently with the approaching pigs. Once finished, the experimenter stood up and tried to have a contact with the remaining pigs as long as they did not escape. This procedure was carried out in each pen of the room. The positive interaction consisted in gently stroking pigs on the nose and behind the ears and, whenever possible, on the back from head to tail in a uniform manner. The NH treatment consisted in entering the room quickly and talking loudly. Inside the pen, after having walked around it and stopped at its centre, the experimenter performed one of the five types of negative interactions with all pigs of the pen. Negative interactions were hurling pressure water with a hose, hurling pressure air with an air gun, loud noise with a horn, immobilization and restraint. To prevent pigs from getting used to negative handling, five different treatments were included and the experimenter did one different negative interaction each day of the week following a random order each week.

At week 27, pigs were transported to the experimental slaughterhouse of IRTA (1.2 km trip), stunned by exposure to 90% CO_2_ at atmospheric air for 3 min and exsanguinated.

### Sample collection

Blood samples from all animals were collected by jugular venepuncture in 10 ml Vacutainer tubes without anticoagulant for serum (Eurotubo^™^, Deltalab, Rubí, Spain) and blood from 16 animals in BD Vacutainer^®^ CPT^™^ tubes (containing sodium heparin anticoagulant with a FICOLL^™^ Hypaque^™^ density fluid) for PBMCs isolation.

PBMCs were isolated following the manufacturer recommendations. Briefly, the tubes were centrifuged at 2000 g for 20 min at room temperature within 2 h of blood collection to avoid alterations of cell properties. The PBMC layer was collected immediately, diluted with 15 ml of PBS buffer (137 mM NaCl, 2.7 mM KCl, 8.1 mM Na_2_HPO_4_·12H_2_O, 1.5 mM KH_2_PO_4_ and pH 7.2–7.4) and centrifuged at 300 g for 15 min at room temperature to obtain the PBMC. Cell pellets were washed with 1 ml of distilled water for 30 s to osmotically lysate the erythrocytes presents in the pellet, and diluted with 15 ml of PBS buffer to stop the osmotic shock. Finally, tubes were centrifuged at 300 g for 15 min at room temperature and rinsed with 15 ml PBS three times to obtain a clean PBMCs pellet and kept frozen at -80°C. Serum from all animals was obtained by centrifugation at 2000 g for 10 min at room temperature. Supernatants were aliquoted and frozen at -80°C until assay.

Saliva samples were collected by allowing pigs chew a cotton swab during approximately 30 s. The saliva was stored in Salivette tubes (Sarstedt, Nümbrecht, Germany) and later centrifuged at 3000 x g for 10 min. Saliva samples were then collected and stored at -80°C until analysis. Hair was collected by shaving the lumbar area of pigs restrained in a snare.

Brains were removed immediately after slaughter and tissue samples from the selected brain structures (amygdala, hippocampus, hypothalamus and prefrontal cortex (PFC)) were excised, collected as quickly as possible in liquid nitrogen and kept frozen at -80°C until analysis.

### Analytical parameters

Serum samples from all animals were analysed for haptoglobin, C-reactive protein (CRP) and Pig-MAP, and glutathione peroxidase (GPx). Haptoglobin was determined spectrophotometrically (Phase Haptoglobin, Tridelta Ltd, County Kildare, Ireland). CRP was determined with an immunoturbidimetric method (OSR #6147, Winston-Salem, NC, USA) validated for porcine samples [35]. Pig-MAP was measured by ELISA (PigChamp ProEuropa, Segovia, Spain). GPx was determined with the Cumene Hydroperoxide method (Ransel, Randox Laboratories Ltd, Crumlin, UK). Superoxide dismutase (SOD) was determined in PBMC lysates with the Xanthine oxidase method (Ransod, Randox Laboratories Ltd, Crumlin, UK). All techniques were adapted to the Olympus AU400 analyser. Cortisol concentrations were determined using commercial ELISA kits, namely DRG Cortisol ELISA (DRG Diagnostics, Marburg, Germany) for serum samples, DRG Salivary Cortisol ELISA (DRG Diagnostics, Marburg, Germany) for salivary samples and High Sensitivity Salivary Cortisol EIA Kit (Salimetrics, State Collage, PA, USA) for hair samples. Protocol for hair extraction and assay validation are described in [36].

### Preparation of PBMCs extracts

PBMC pellets were suspended with 500 μl of HBSS solution (PBS buffer and 5.55 mM D-glucose) with protease inhibitors (protease inhibitors cocktail, Sigma-Aldrich, St. Louis, MO) and sonicated twice at 30% amplitude for 10 s on ice (Branson Digital Sonifier, model 250, Branson Ultrasonics Corp., Danbury, CT). These extracts were used for SOD assay.

For immunoblot and proteomic studies, the PBMC extracts were desalted and precipitated using 2D Clean-Up Kit (GE Healthcare, Buckinghamshire, UK). Protein pellets were suspended with a volume of IEF sample buffer (7 M urea, 2 M thiourea, 4% (w/v) CHAPS and pH 8.5) and protein concentration was quantified using RcDc Protein Assay Kit (Bio-Rad, Hercules, CA). Finally, protein extracts from PBMC lysates were frozen at -80°C until DIGE analysis.

### Immunoblotting

Slot blot was performed for detection of carbonyl groups contained in proteins [37]. Protein extracts from PBMC (0.5 μg protein) were applied to each slot and transferred for 20 min onto PVDF membrane (Immuno-Blot PDVF, Bio-Rad, Hercules, CA). Proteins in the membrane were derivatized by incubating the membrane in 0.5 M 2,4-dinitrophenylhydrazine (DNPH, Sigma, St. Louis, MO) for 5 min. After washing, the membranes were blocked with 5% skim milk in TBS-T solution (20 mM Tris-HCl, 150 mM NaCl, 0.05% Tween 20 and pH 7.4) and incubated overnight at 4°C with anti-DNP antibody (Anti-dinitrophenyl (DNP), Sigma (St. Louis, MO)). Membranes were incubated with horseradish peroxidase (HRP)-conjugated immunoglobulin (Goat anti-rabbit IgG (H+L) HRP-linked antibody, Cell Signalling, Danvers, MA) and visualized by chemiluminescence (ECL, GE Healthcare, Buckinghamshire, UK). Image densitometry was performed using Multi Gauge software (Fujifilm, Tokyo, Japan). A control serum sample was used in all the immunoassays in order to compare results from different membranes.

For western blot, PBMC protein extracts (9 μg protein) were resolved in SDS-PAGE under reducing conditions (10–15% acrylamide) and immunoblotted with polyclonal antibodies against MYLC2 (Cell Signaling, Danvers, MA), fibrinogen (Sigma, St. Louis, MO) and β-actin (Santa Cruz, Santa Cruz, CA) as loading control.

### Differential gel electrophoresis (DIGE) and protein identification by mass spectrometry (MS)

#### DIGE

A total of 16 PBMC individual samples (8 animals randomly chosen, t0 and t2 month, 4 animals each handling group) were used in this experiment, without pooling. Using pH strips, pH of protein extracts from PBMC lysates was adjusted at 8.5. Then 50 μg of protein from protein extracts were labelled with Cy3 or Cy5 cyanine dyes (400 pmol dye/50 μg protein) (GE Healthcare, Buckinghamshire, UK) for 30 min on ice in dark and reaction was quenched with 10 mM lysine for 10 min. Dye-swap was performed to avoid possible bias introduced by labelling efficiency. A pool of protein extracts from all animals and times (50 μg total protein) was labelled with Cy2 as internal standard.

Samples were combined according to experimental design at 50 μg protein/Cy/gel and diluted with a volume of IEF sample buffer adding 2% (w/v) DTT, 2% (v/v) ampholytes pH 3–10 and 0.002% (v/v) Bromophenol blue. IPG strips (24 cm, linear gradient pH 3–10, GE Healthcare) were rehydrated overnight with IEF buffer and first-dimension was performed on an Ettan IPGphor system (GE Healthcare, Buckinghamshire, UK) for a total of 68 kV/h. The strips were equilibrated for 15 min in a reducing solution (6 M urea, 100 mM Tris-HCl pH 8, 30% (v/v) glycerol, 2% (w/v) SDS, 5 mg/mL DTT and 0.002% (w/v) bromophenol blue), and incubated in an alkylating solution (6 M urea, 100 mM Tris-HCl pH 8, 30% (v/v) glycerol, 2% (w/v) SDS, 22.5 mg/mL iodoacetamide, and 0.002% (v/v) bromophenol blue) for 15 min.

Second-dimension was performed using eight SDS-PAGE reducing gels (12.5% acrylamide, 24 x 20 cm) on an EttanDALT system (GE Healthcare, Buckinghamshire, UK). Finally, gels were scanned on a Typhon 9400 system (GE Healthcare, Buckinghamshire, UK) at 488 nm/520 nm for Cye2, 532 nm/580 nm for Cye3 and 633 nm/670 nm for Cye5 excitation/emission wavelengths respectively, at a 100 μM resolution.

Gels images were analysed and statistically quantified using Progenesis SameSpots v4.5 (NonLinear Dynamics, Newcastle, UK) software. Analysis of variance (ANOVA) was applied to matched spots and the data was filtered to retain spots with ANOVA p values of 0.05 or less.

#### Protein identification by mass spectrometry

A total of 64 spots were selected on the basis of an ANOVA test *P* < 0.05 and >1.5 fold volume difference and digested with trypsin [38]. Only proteins exhibiting a fold change greater than the median fold change for increased and decreased proteins were selected. Dried peptides were resuspended and analysed on an LTQ Velos-Orbitrap mass spectrometer (ThermoFisher Scientific, Bremen, Germany), coupled to a nano-HPLC system (Proxeon, Odense, Denmark). MS/MS fragmentation spectra (200 ms, 100–2800 m/z) of 20 of the most intense ions as detected from a 500 ms MS survey scan (300–1500 m/z), were acquired using a dynamic exclusion time of 20 s for precursor selection and excluding single-charged ions. Precursor scans were acquired in the Orbitrap analyser at a mass resolution of 30000. MS/MS spectra were acquired at the LTQ Velos analyser, using a relative collision energy of 35. An intensity threshold of 1000 counts was set for precursor selection.

Searches were performed using the software suite ProteinScape 3.1 (Bruker, Bremen, Germany), from mascot generic files, mgf, generated from the raw data using Proteome Discoverer 1.4 software (ThermoFisher, Bremen, Germany). MS/MS spectra were searched with a precursor mass tolerance of 10 ppm (Orbitrap measurements were performed enabling the lock mass option (m/z 445.120024) to improve mass accuracy), fragment tolerance of 0.5 Da and trypsin specificity, allowing for up two missed cleavages. Cysteine carbamidomethylation and methionine oxidation were set as fixed and variable modifications, respectively. Searches were performed against a proteome database constructed with the pig sequenced proteins present in the UniProt database ((http://www.uniprot.org/).

#### Bioinformatic analysis

Proteins names identified by mass spectrometry were introduced on PANTHER version 9.0 software (http://pantherdb.org/) for Gene Ontology (GO) classifications, together with UniProt databases (http://www.uniprot.org/). For proteins interaction network analysis, identified proteins were analysed with STRING version 10 (http://string-db.org/) and network interaction was represented.

### Quantification of brain neurotransmitters

Brain areas samples from all animals were weighted and homogenized (1:10 w/w) in an ice-cold 0.25 M perchloric acid containing 0.1 M Na_2_S_2_O_5_ and 0.25 M EDTA. Dihydroxybenzylamine (DHBA) and Nω-metil-5-hydroxytryptamine (Nω) were added as internal standards for catecholamines and indoleamines, respectively. The mixture was homogenized by sonication (Branson Digital Sonifier, model 250, Branson Ultrasonics Corp., Danbury, CT) followed by centrifugation at 3000 g for 10 min at 4°C and the supernatant were kept frozen at -80°C. After centrifugation at 12000 g for 10 min at 4°C, concentrations of noradrenaline (NA), dopamine (DA), 3,4-dihydroxyphenylacetic acid (DOPAC), homovanillic acid (HVA), serotonin (5-HT) and 5-hydroxyindole-3-acetic acid (5-HIAA) were determined by HPLC (Elite LaCHrom, Merck, Hitachi, Japan) equipped with a Cromolith Rp-18e column (Merck KgaA, Darmstadt, Germany) with electrochemical detection (ESA Coulochem II 5200, Bedford, MA). Injected volume was 20 μL for all samples. The mobile phase consisted of 0.5 M citrate buffer pH 2.8, 0.05 mM EDTA, 1.2 mM sodium octyl sulphate (SOS) and 1% acetonitrile. The applied voltage was set at 400 mV for electrochemical detection and the flow rate was 1 ml/min.

The chromatographic quantification of NA, dopaminergic and serotonergic NTs showed a good precision, with coefficient of variation between-days and within-days lower than 4%. Linearity was evaluated between 2.5–80 pg/μl for 5-HT, 5–160 pg/μl for Nω, 5–240 pg/μl for HVA and 2.5–120 pg/μl for the rest of NTs. Coefficients of determination (R2) were calculated and found to be higher than 0.999 for all analytes. Limit of detection was between 2.14 and 4.97 pg/μL and the limit of quantification was between 6.48 and 15.06 pg/μL for all the analytes. The internal control DHBA allowed the comparison between runs. Dopaminergic total sytem (DA-system) and serotonergic total system (5-HT-system) are calculated as the sum of all metabolites in the pathway (DA, DOPAC and DA; and 5-HT and 5-HIAA; respectively). Noradrenergic system (NA-system) is only composed by NA concentration.

### Statistical analysis of biochemical and NT data

The Statistical Analyses System (SAS v9.4; software SAS Institute Inc., Cary, NC; 2002–2008) was used to analyse data. Descriptive data is presented with the means and the standard error and the significance level was established at P < 0.05 and a tendency was considered at 0.05 ≥ P ≤ 0.1.

Normality test of data and residuals was performed for each measure. Whenever possible, data was log transformed to correct the distribution.

The MIXED procedure with repeated measures analysis was performed for biochemical data. The full factorial model includes time (t0 and t2) as within-subject factor, handling (PH or NH) as between-subject factor and their interaction. Pig was introduced as the experimental unit and the housing pen as a random effect nested within the two handling treatments.

MIXED procedure with Tukey adjustment was performed for NT and oxidative markers data. Each pig was introduced as the experimental unit, handling (PH or NH) as fixed effect and the housing pen as a random effect nested within the two handling treatments.

## Results

### Concentration of cortisol, acute phase proteins and GPx

Cortisol was determined in serum, saliva and hair in all individuals under both handling conditions at each time point (t0 and t2) (Table 1). Hair cortisol decreased at t2 *vs* t0 and there was a tendency for hair and serum cortisol depending on handling. No differences were observed in saliva cortisol and no interaction between both factors in any of the measurements. Serum acute phase proteins (haptoglobin, CRP (*P* < 0.001) and Pig-MAP (*P* < 0.1)) decreased significantly at t2, whereas the antioxidant enzyme GPx increased at t2 (*P* < 0.001) (Table 1). There was no effect of handling, and there was no interaction between time and handling for any variable.

**Table 1:** Cortisol, acute phase proteins and GPx in pigs subjected to PH and NH.

	t 0				t 2				Handling	Time	Handling*Time
	NH		PH		NH		PH				
	Mean	SE	Mean	SE	Mean	SE	Mean	SE	*P*	*P*	*P*
Hair Cortisol (pg/mg)	24.70	2.05	19.80	1.35	20.80	2.00	17.61	1.53	0.208	0.007	0.852
Serum Cortisol (ng/mL)	23.50	2.22	17.57	1.77	24.21	2.09	21.20	2.09	0.094	0.252	0.410
Saliva Cortisol (ng/mL)	3.47	0.30	4.25	0.57	3.57	0.21	3.60	0.27	0.604	0.172	0.826
Haptoglobin (mg/mL)	0.64	0.07	0.86	0.07	0.39	0.06	0.43	0.04	0.166	<0.0001	0.047
CRP (μg/dL)	16.81	1.29	19.19	1.79	9.23	1.36	8.64	0.91	0.946	<0.0001	0.218
Pig-MAP (mg/mL)	0.98	0.10	1.00	0.10	0.80	0.08	0.92	0.12	0.764	0.021	0.588
GPx (U/L)	6067	214	6149	188	7944	302	7841	246	0.975	<0.0001	0.475

### Proteomic analysis of PBMCs

**Fig 2:**
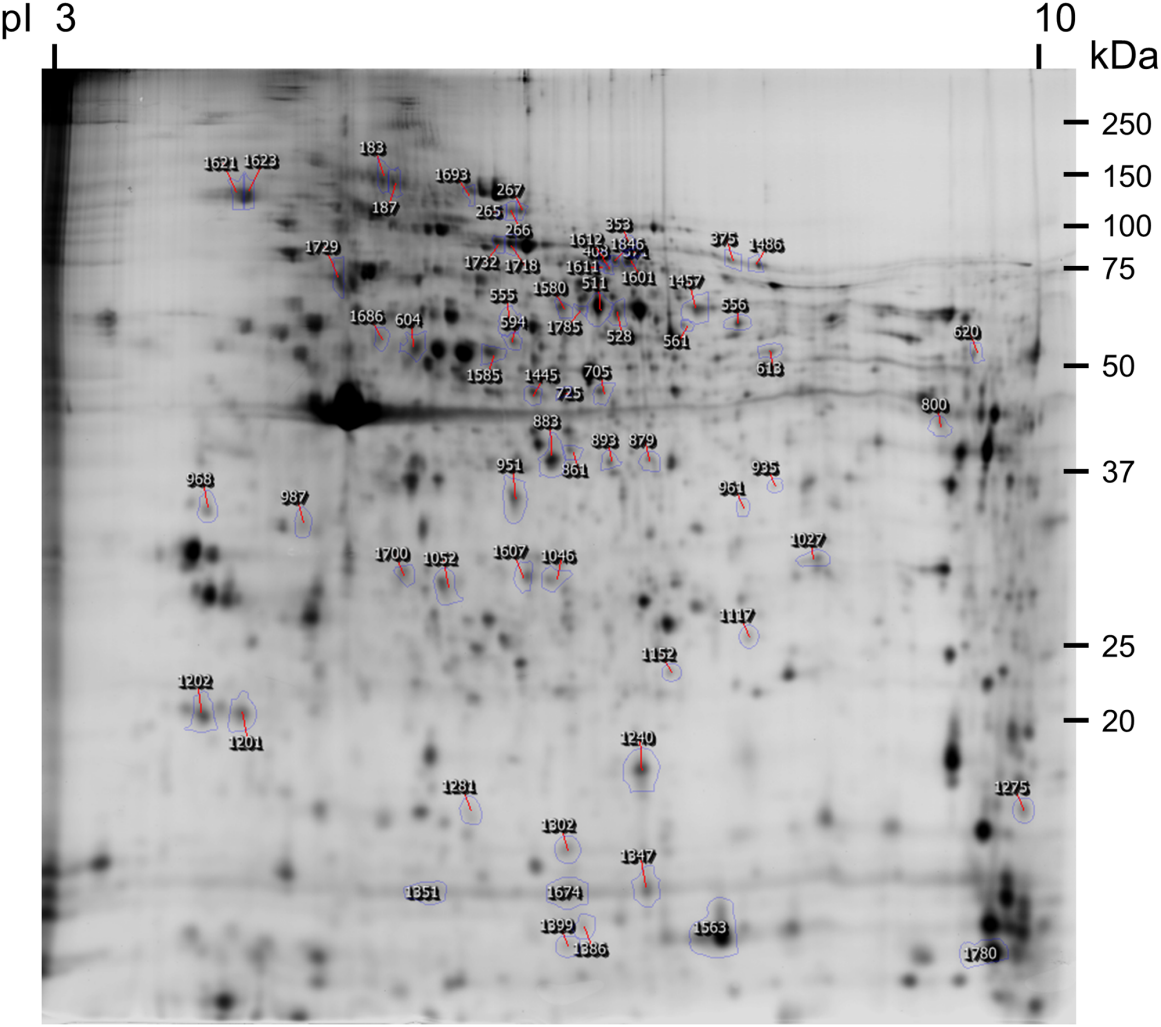
Master gel from porcine PBMC. The position of the differentially abundant spots that were identified is shown.

The total PBMC proteome was analysed by DIGE. A representative gel is shown in Fig 2. Many of the proteins identified in the present study have been already identified in porcine PBMCs [25]. The 2-DE map obtained in this study was very similar to that reported by these authors with actin as the main spot in the gel (MW ~ 44 kDa, pI ~ 5.7).

A total of 1180 spots were analysed. Comparing differences in protein abundance between t0 and t2 months, 305 spots were localized in the NH group and 153 spots in the PH group with the defined criteria (*P* < 0.05 and >1.5 fold change difference).

Sixty-four differential spots were selected for mass spectrometry analysis on basis of the spot form, distribution and discarding those placed in the side borders of the gel. A total of 54 different proteins of the 64 selected spots were identified by LC-MS/MS (S1 and S2 Tables).

Thirty-seven spots were upregulated at t2 versus t0 whereas 27 were downregulated. In most of the cases the fold-change variation between t0 and t2 was larger in the NH group (54 protein spots) than in the PH group (10 protein spots). Six proteins were altered only in the NH group. No protein was found differentially expressed only in the PH group. Results are presented in Table 2. Only three proteins were differently abundant between NH and PH groups at t0 (KHSRP, PSMA4, and caldesmon) and one at t2 (CCT2).

**Table 2:** List of proteins differentially expressed in PBMC at t2 versus t0 in PH and NH groups. Proteins with a Fold-change ≥ 1.5 and *P* < 0.05 in at least one of the comparisons are included. The ratio between NH t2/t0 and PH t2/t0 is shown to visualize which group shows the larger variation. ND: not determined if one of the groups does not comply with the criteria of Fold-change ≥ 1.5 and *P* < 0.05. NR: Not represented on STRING.

					NH		PH		
					t2/t0		t2/t0		
Spot	Identification UniProt Pig	Gene	Accession Number UniProt Pig	STRING Name	Fold	*P*	Fold	*P*	Fold-change Ratio NH/PH
Group A									
861	Annexin	ANXA1	F1SJB5_PIG	ANXA1	-4.1	0.004	-2.8	0.0003	1.46
	Leukocyte elastase inhibitor	SERPINB1	ILEU_PIG	SERPINB1					
883	Annexin	ANXA1	F1SJB5_PIG	ANXA1	-5.7	0.002	-2.7	0.049	2.11
987	EF-hand domain-containing protein D2	EFHD2	F1SUW3_PIG	EFHD2	1.8	0.042	2.1	0.026	0.86
1046	High mobility group protein B1	HMGB1	F2Z594_PIG	HMGB1	-2.2	0.002	-2.4	0.018	0.92
1240	Calcium-binding protein A9	S100A9	C3S7K6_PIG	S100A9	-11.8	0.0007	-4.3	0.025	2.74
1275	Peptidyl-prolyl cis-trans isomerase A	PPIA	PPIA_PIG	PPIA	-2.1	0.046	-1.7	0.036	1.24
1607	High mobility group protein B1	HMGB1	F2Z594_PIG	HMGB1	-1.9	0.006	-2	0.014	0.95
1729	Plastin-2	LCP1	F1RK02_PIG	LCP1	-2.9	0.01	-2	0.016	1.45
Group B									
183	Integrin alpha-IIb	ITGA2B	F1RQY8_PIG	ITGA2B	2.1	0.003	1.6	0.027	1.31
187	Integrin alpha-IIb	ITGA2B	F1RQY8_PIG	ITGA2B	2.6	0.017	1.7	0.024	1.53
265	Filamin-A	FLNA	Q2YHQ3_PIG	FLNA	4.2	0.0007	3.3	0.008	1.27
267	Filamin-A	FLNA	Q2YHQ3_PIG	FLNA	3.5	0.003	2.8	0.015	1.25
408	Coronin 1 C	CORO1C	F1RGA9_PIG	CORO1C	2.4	9.6E-05	4.3	0.022	0.56
511	Fibrinogen Beta Chain	FGB	F1RX37_PIG	FGB	3.9	0.007	2.1	0.041	1.86
528	Fibrinogen Alpha Chain	FGA	F1RX36_PIG	FGA	3	0.001	2	0.025	1.50
555	Coronin 1 A	CORO1A	G8G223_PIG	CORO1A	-2	0.003	-1.4	0.035	ND
556	Fibrinogen beta chain	FGB	F1RX37_PIG	FGB	3.9	0.012	1.9	0.044	2.05
594	Coronin 1A	CORO1A	I3LR17_PIG	CORO1A	-2	0.047	-1.4	0.032	ND
604	Fibrinogen gamma chain	FGG	F1RX35_PIG	FGG	3	0.001	2	0.046	1.50
613	Integrin-linked protein kinase	ILK	I3L9C8_PIG	ILK	4.5	0.01	2.6	0.025	1.73
620	Testin	TES	TES_PIG	TES	1.5	0.046	2.4	0.027	0.63
705	Pleckstrin	PLEK	F1SJ07_PIG	PLEK	2.6	0.005	2.1	0.019	1.24
725	Pleckstrin	PLEK	F1SJ07_PIG	PLEK	2.4	0.0005	2	0.042	1.20
	Alpha-centractin	ACTR1A	F2Z5G5_PIG	ACTR1A					
879	PDZ and LIM domain protein 1	PDLIM1	T1RTP3_PIG	PDLIM1	1.5	0.009	2	0.048	0.75
893	PDZ and LIM domain protein 1	PDLIM1	T1RTP3_PIG	PDLIM1	3.4	0.006	4.1	0.011	0.83
935	Calponin-2	CNN2	CNN2_PIG	CNN2	2.2	0.008	1.7	0.019	1.29
961	Calponin-2	CNN2	CNN2_PIG	CNN2	-4.4	2.8E-05	-3.8	0.007	1.16
968	Tropomyosin alpha-1 chain	TPM1	TPM1_PIG	TPM1	2.4	0.04	1.7	0.03	1.41
1201	Myosin regulatory light polypeptide 9	MYL9	MYL9_PIG	MYL9	4	0.01	2.5	0.032	1.60
1202	Myosin regulatory light chain 12B	MYL12B	F1SM78_PIG	MYL12B	2.1	0.006	1.6	0.031	1.31
1399	Filamin-A	FLNA	Q2YHQ3_PIG	FLNA	-2.6	0.011	-1.7	0.022	1.53
1445	Pleckstrin	PLEK	F1SJ07_PIG	PLEK	5.7	0.0008	3.4	0.026	1.68
1457	Fibrinogen beta chain	FGB	F1RX37_PIG	FGB	3.5	0.0005	2.5	0.014	1.40
1580	Fibrinogen beta chain	FGB	F1RX37_PIG	FGB	3.1	0.001	2.2	0.042	1.41
1585	Fibrinogen gamma chain	FGG	F1RX35_PIG	FGG	3	0.007	1.8	0.02	1.67
1601	Fibrinogen alpha chain	FGA	Q28936_PIG	FGA	9.4	0.005	8	0.003	1.18
1611	WD repeat-containing protein 1	WDR1	K9IVR7_PIG	WDR1	3.3	0.004	6.7	0.017	0.49
1612	Fibrinogen alpha chain	FGA	F1RX36_PIG	FGA	8.6	0.015	6.4	0.004	1.34
1621	von Willebrand factor	VWF	F5XVC2_PIG	VWF	4.1	0.016	2.2	0.021	1.86
1623	Endoplasmin	HSP90B1	ENPL_PIG	HSP90B1	2.7	0.018	2	0.004	1.35
1693	Vinculin	VCL	VINC_PIG	VCL	5	0.003	2.7	0.023	1.85
1718	Coagulation factor XIII, A1 polypeptide	F13A1	K7GQL2_PIG	NR	2.6	0.009	1.6	0.047	1.63
1732	Caldesmon	CALD1	F1SNH3_PIG	CALD1	2.5	0.014	1.6	0.036	1.56
1785	WD repeat-containing protein 1	WDR1	K9IVR7_PIG	WDR1	2.7	0.029	1.7	0.015	1.59
	Fibrinogen beta chain	FGB	F1RX37_PIG	FGB					
Group C									
353	Far upstream element-binding protein 2	KHSRP (FUBP2)	F1SBT6_PIG	KHSRP	-3.4	0.001	-1.2	0.23	ND
561	tRNA-splicing ligase RtcB homolog	RTCB	RTCB_PIG	C22orf28	-2	0.003	-1.4	0.018	ND
800	Elongation factor 1-alpha	EEF1A1	Q0PY11_PIG	EEF1A1	-2.3	0.007	-1.5	0.031	1.53
961	Heterogeneous nuclear ribonucleoprotein A2/B1	HNRNPA2B1	M3UZ37_PIG	HNRNPA2B1	-4.4	2.8E-05	-3.8	0.007	1.16
968	Heterogeneous nuclear ribonucleoprotein K	HNRNPK	I3LQS0_PIG	HNRNPK	2.4	0.04	1.7	0.03	1.41
1117	Heterogeneous nuclear ribonucleoprotein A2/B1	HNRNPA2B1	M3UZ37_PIG	HNRNPA2B1	-5.6	0.044	-2.9	0.014	1.93
1152	Heterogeneous nuclear ribonucleoprotein A2/B1	HNRNPA2B1	M3UZ37_PIG	HNRNPA2B1	-11.6	0.048	-5.2	0.002	2.23
1281	GTP-binding protein Rheb	RHEB	F2Z5R2_PIG	RHEB	-1.9	0.022	-2.3	0.005	0.83
1347	Histone H2B	HIST1H2BF	F2Z580_PIG	HIST1H2BF	-1.9	0.009	-2.3	0.0002	0.83
1351	Histone H2B	HIST1H2BF	F2Z580_PIG	HIST1H2BF	-1.8	0.014	-2.6	0.006	0.69
1611	Far upstream element-binding protein 1	FUBP1	F1S9S5_PIG	FUBP1	3.3	0.004	6.7	0.017	0.49
1674	Histone H2B	HIST1H2BF	F2Z580_PIG	HIST1H2BF	-2.4	0.008	-2.4	0.0001	1.00
Group D									
594	Chaperonin containing TCP1, subunit 2 (Beta)	CCT2	D0G0C8_PIG	CCT2	-2	0.047	-1.4	0.032	ND
1027	Proteasome subunit alpha type-4	PSMA4	F2Z528_PIG	PSMA4	2.2	0.009	1.4	0.016	ND
1052	Proteasome activator complex subunit 1	PSME1	PSME1_PIG	PSME1	-2.2	0.0005	-1.7	0.034	1.29
1275	SUMO-conjugating enzyme UBC9	UBE2I	I3LSZ1_PIG	UBE2I	-2.1	0.046	-1.7	0.036	1.24
1700	Proteasome activator complex subunit 2	PSME2	PSME2_PIG	PSME2	-2.9	0.001	-2.2	0.008	1.32
Group E									
594	Cytosol aminopeptidase	LAP3	I3LD43_PIG	LAP3	-2	0.047	-1.4	0.032	ND
1302	Peroxiredoxin-5, mitochondrial	PRDX5	F1RQP0_PIG	PRDX5	-2.6	0.005	-1.8	0.016	1.44
1686	Cytosolic non-specific dipeptidase	CNDP2	F1SNL7_PIG	CNDP2	-2.3	0.003	-2.3	0.008	1.00
Group F									
528	GMP synthase	GMPS	I3LJ73_PIG	GMPS	3	0.001	2	0.025	1.50
705	Small calcium-binding mitochondrial carrier 1	SLC25A24	B2MUB6_PIG	SLC25A24	2.6	0.005	2.1	0.019	1.24
935	Monoglyceride lipase	MGLL	B8XSJ9_PIG	MGLL	2.2	0.008	1.7	0.019	1.29
951	Malate dehydrogenase, cytoplasmic	MDH1	MDHC_PIG	MDH1	-2.3	0.002	-1.6	0.031	1.44
1386	Histidine triad nucleotide-binding protein 1	HINT1	F1RKI3_PIG	HINT1	-2.8	0.011	-1.9	0.026	1.47
1486	Aconitate hydratase, mitochondrial	ACO2	F1SRC5_PIG	ACO2	-2	0.028	-1.5	0.044	1.33

### Functional analysis of identified proteins

**Fig 3:**
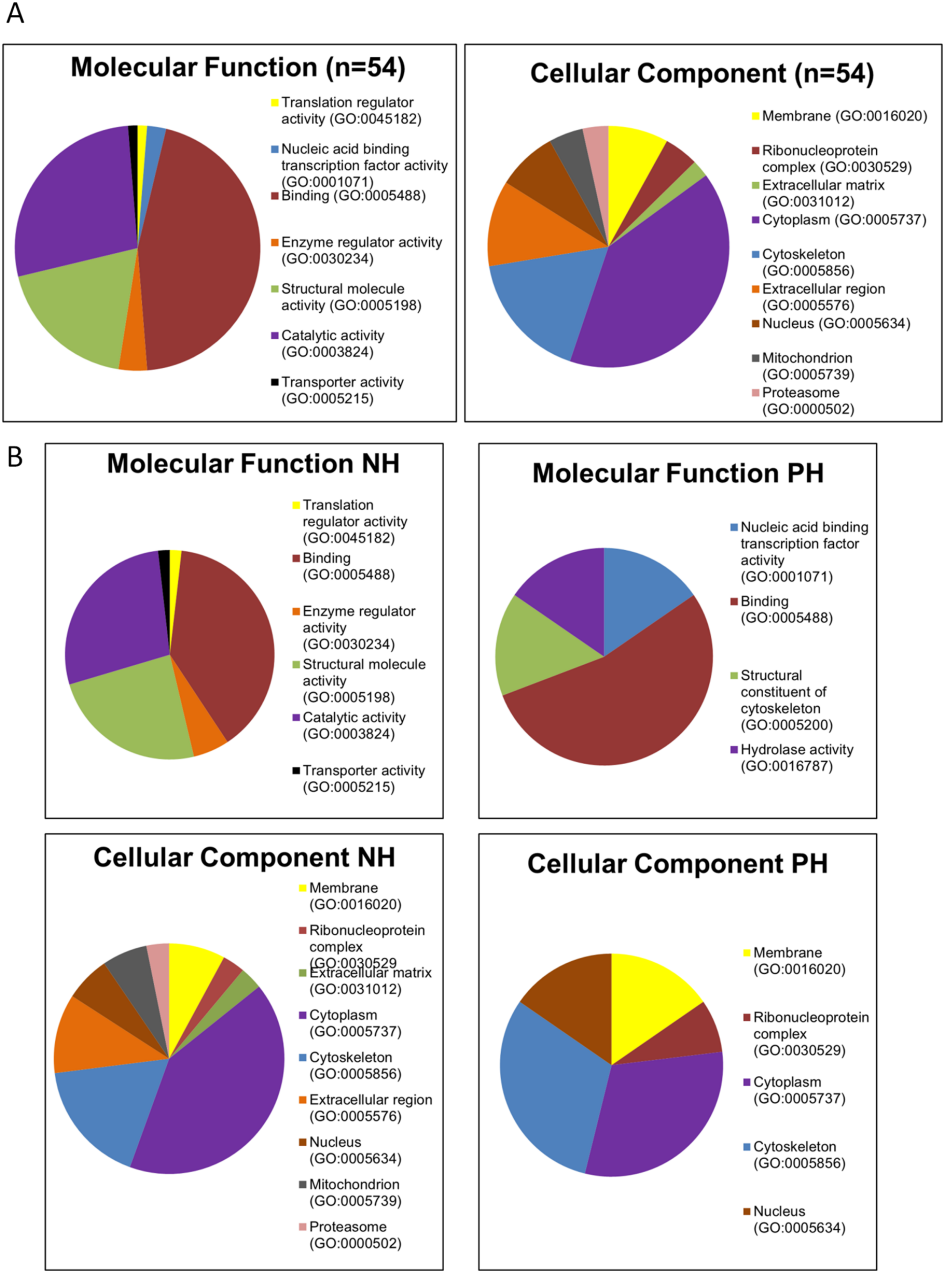
Gene Ontology analysis of proteins identified by DIGE with fold change ≥1.5 and *P*<0.05 criteria, classified as molecular function and cellular component. A) Global analysis of GO categories. Molecular function defined mainly binding proteins (40%) mostly related to protein-protein interaction, structural proteins involved in cell cytoskeleton (23%) and proteins with catalytic activity (28%), specifically hydrolase activity. Regarding cell component, cytoplasm (40%) and cytoskeleton (17%) were predominant. B) GO categories as displayed in NH and PH groups.

Gene Ontology (GO) analysis is shown in Fig 3A. The GO analysis of the proteins identified in NH and PH groups, clearly revealed that the NH group had more GO terms and greater percentage of annotations compared to the PH group (Fig 3B).

**Fig 4:**
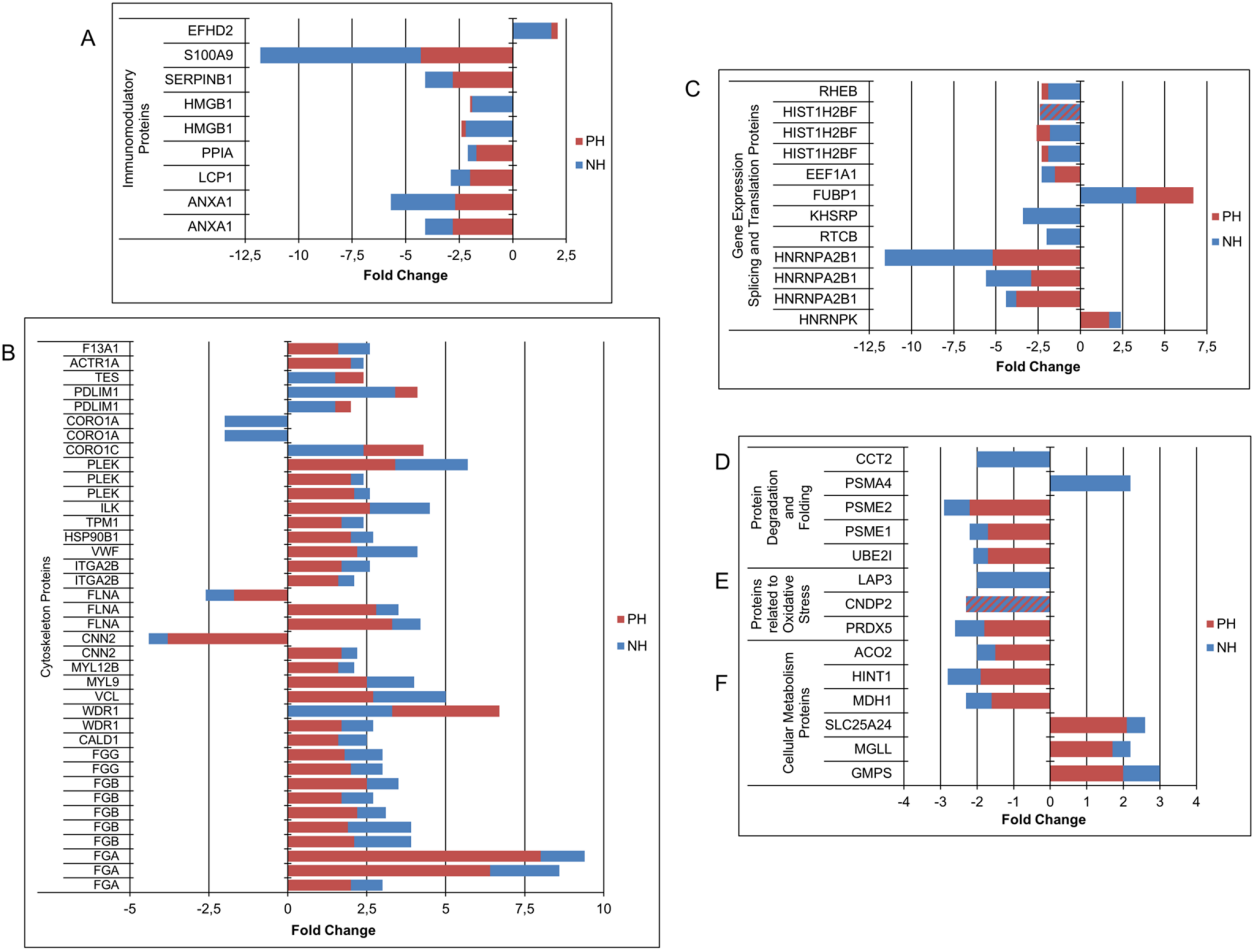
Changes in protein abundance classified by their biological function. Horizontal axes represent fold change (≥1.5 and *P*<0.05) in NH (blue) and PH (red) groups over time.

Biological processes illustrated in Fig 4 are classified in six groups: A) Immunomodulatory proteins, including Inflammatory response (GO:00006954), Innate immune response (GO:0045087), Viral process (GO:0016032), T cell activation involved on immune response (GO:0002286); B) Cytoskeleton proteins, including specific GO: Cell-matrix adhesion (GO:0007160), Positive regulation of actin depolymeration (GO:0030836), Movement of cell component (GO:0006928), Regulation of cell shape (GO:0008360), Actomyosin structure organization (GO:0031032), Extracellular matrix organization (GO:0030198), Actin rod assembly (GO:0031247), Cytoskeleton organization (GO:0007010), Integrin-mediated signalling pathway (GO:0007229); C) Gene expression, splicing and translation proteins, including Transcription regulation (GO:0006355), tRNA splicing (GO:0008033), Gene expression (GO:0010467); D) Protein degradation and folding, including Protein polyubiquitination (GO:0000209), Regulation of proteasomal protein catabolic process (GO:0061136), Protein folding (GO:0006457); E) Proteins related to oxidative stress, including Glutathione biosynthetic process (GO:0006750), Response to oxidative stress (GO:0006979) and F) Cellular metabolism proteins, including TCA cycle (GO:0006099), Purine nucleotide biosynthetic process (GO:0006164), Fatty acid biosynthetic process (GO:0006633).

**Fig 5:**
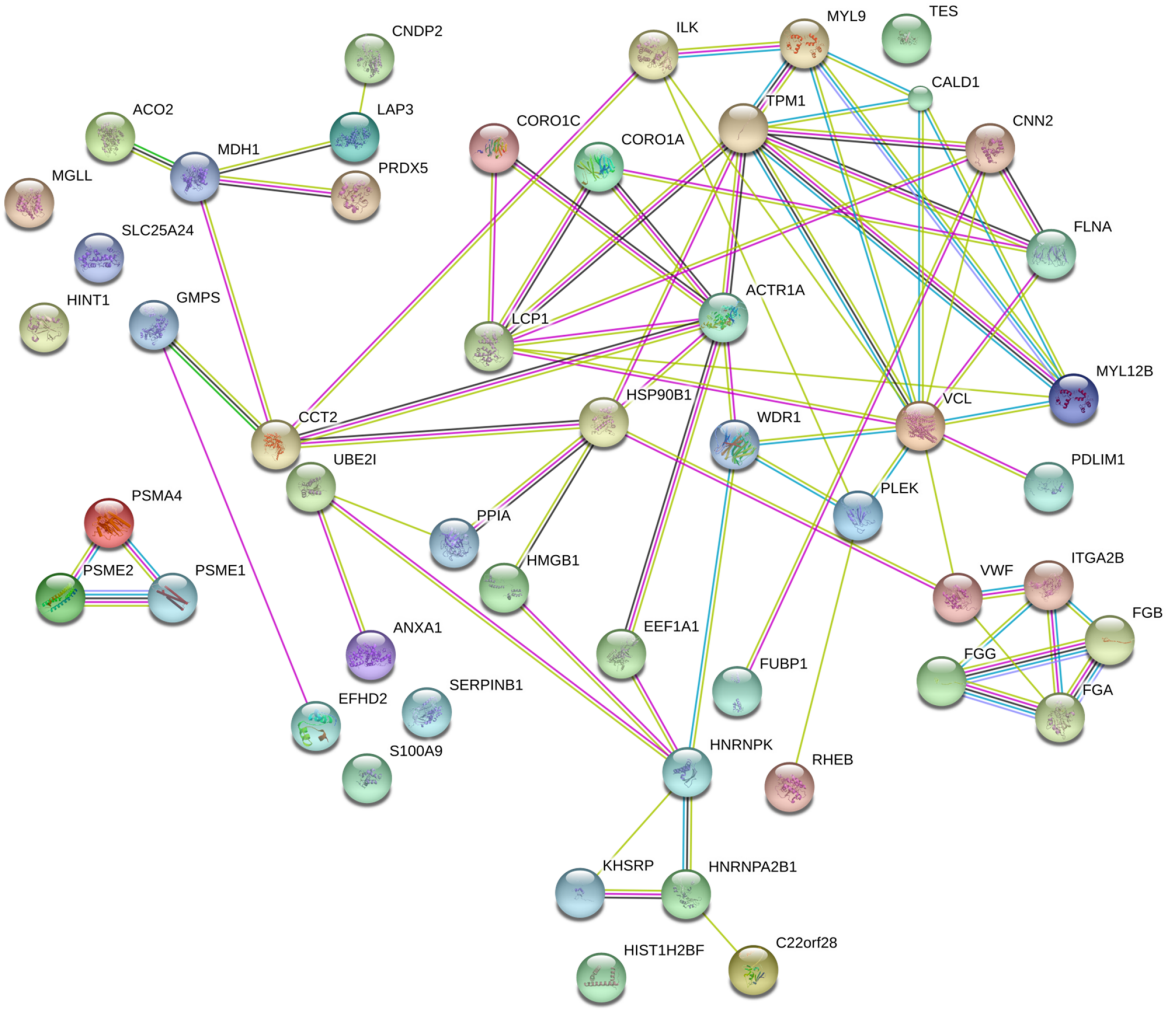
STRING network of 54 proteins identified by DIGE. Links between nodes represent different types of evidence for association (Yellow = Text mining, Pink = Experiments, Cian = Databases, Black = Coexpression, Green = Neighbourhood, Grey = Homology).

All the identified proteins were analysed with the STRINGv10 software for functional networks. Nodes and interaction between identified proteins are shown in Fig 5.

### Validation of handling-related proteins

**Fig 6:**
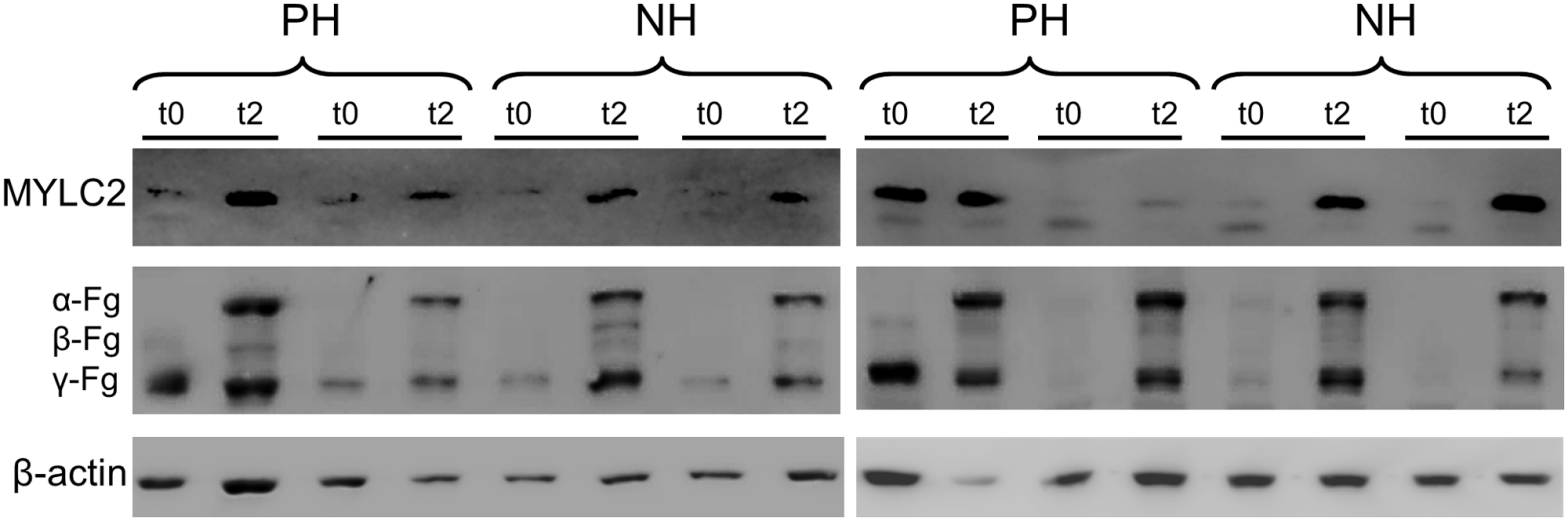
Western blot of myosin light chain 2 (MYLC2) and fibrinogen (chains α, β, γ) from PBMCs from the individuals included in the DIGE study at t0 and t2 in NH and PH groups. β-actin was used as loading control.

Two proteins, myosin-light chain (MYLC2) and fibrinogen (FG) were selected to validate their intrinsic expression using immunoblot analysis with antibodies specific for porcine proteins. Western blot analysis corroborated that both proteins increased at t2 versus t0 (Fig 6).

### Markers of oxidative stress

**Fig 7:**
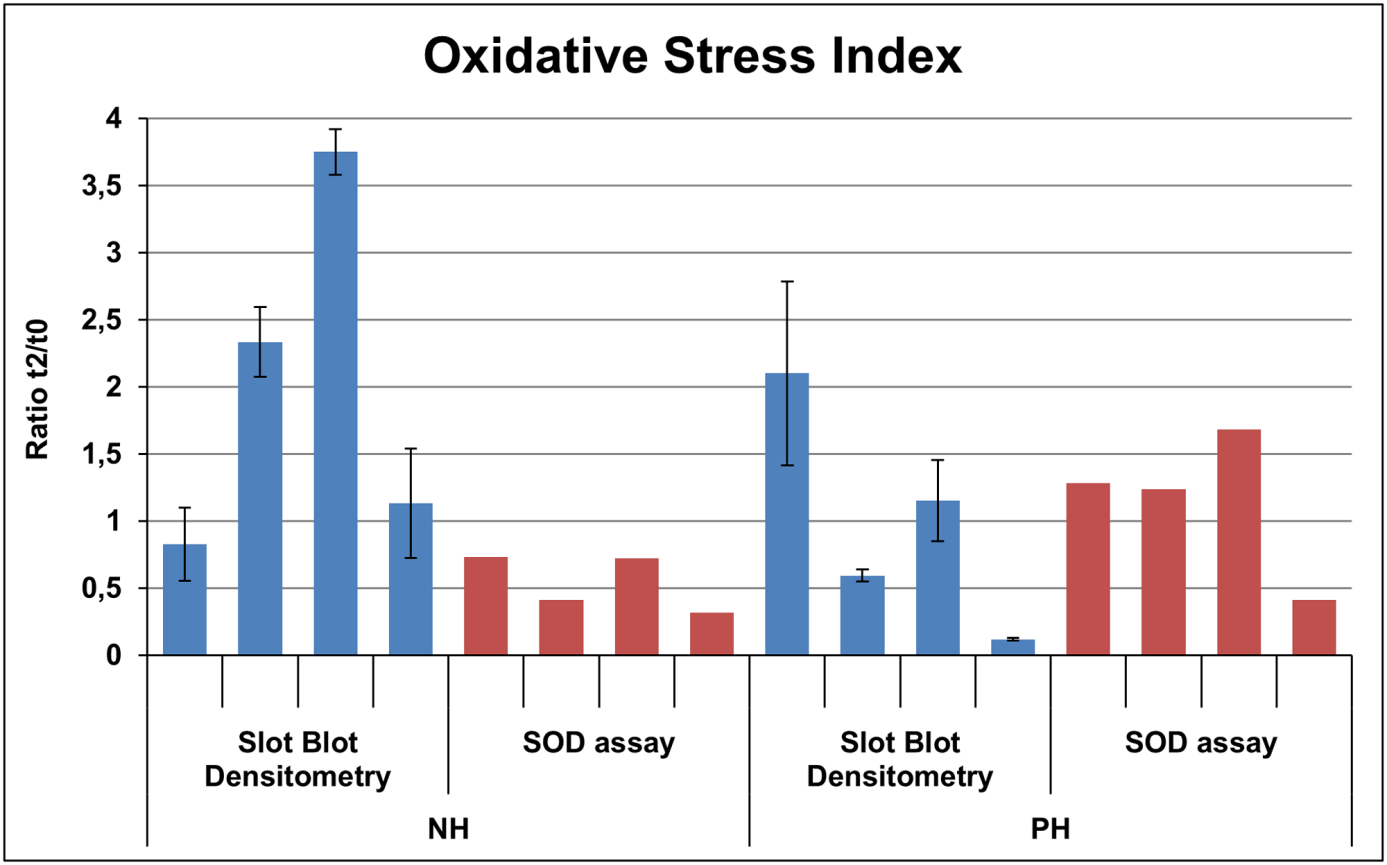
Protein carbonylation (blue) and SOD activity (red) in PBMC extracts from the eight individuals included in the DIGE study. The ratio between t2 and t0 is shown. Error bars correspond to the SE from three independent replicates for each assay.

Since the bioinformatic analysis of data revealed alterations in redox metabolism, and serum GPx activity increased at t2, other markers of oxidative stress were analysed in PBMC extracts from the individuals included in the DIGE experiment: total protein carbonylation and SOD activity, measured as the ratio of oxidized proteins and the ratio of SOD between t2 and t0. As shown in Fig 7, three of the PH-pigs and only one NH-pig showed a SOD activity ratio higher than 1, whereas one PH-pig and two NH-pig showed a protein carbonylation lower than 1.5. Due to the high individual variability, statistical analysis showed no significance for the ratio of oxidized proteins (*P* = 0.244) and a tendency for the ratio of SOD (*P* = 0.078).

### Neurotransmitter profile

Neurotransmitters from the catecholamine and serotonin pathways in four brain areas (amygdala, hippocampus, PFC and hypothalamus) were analysed. Statistical tendencies were observed in the concentration of serotonin (5-HT), which increases in the PFC in animals with PH, whereas it decreases in the amygdala in animals in the same condition (Table 3).

**Table 3:** Brain serotonin (5-HT) concentration (ng/g tissue) in the prefrontal cortex and amygdala of pigs subjected to PH or NH.

*Brain area*	*NH*		*PH*		*P*
	*Mean*	*SE*	*Mean*	*SE*	
Prefrontal cortex	200.45	12.90	229.18	10.19	0.093
Amygdala	683.86	22.46	627.61	20.90	0.073

## Discussion

The stress degree in the farm after mixing of the animals and their subsequent adaptation to the farm was assessed by analysing the concentration of cortisol in serum, saliva and hair at both time points. Hair cortisol showed a significant decrease, indicating that environmental stress decreased throughout time. The deposition of cortisol in hair is considered a good marker of chronic stress in several animal species [39–41]. The decrease in hair cortisol was not accompanied by changes in cortisol in serum and saliva, but this is not unexpected since serum and salivary cortisol are markers of acute stress and display high biological variability. Acute phase proteins (haptoglobin, CRP and Pig-MAP) decreased throughout the experimental period. APPs are markers of inflammation, but they also increase in stress situations [11–13,42]. Taken together, these results indicate that the animals had suffered stress at the beginning of the experiment, probably associated to group mixing on farm, but later they adapt to the farm environment [43]. There were no differences in hair, serum and saliva cortisol between handling treatments.

Several sets of proteins were differentially regulated over time in PH and NH conditions, which are shown grouped by their biological function in Fig 4. The figure clearly shows that these sets of proteins are altogether up- or downregulated between t0 and t2. Many of the identified proteins share the characteristic of being potentially regulated by cortisol, indicating that changes in protein abundance between t0 and t2 are, at least in part, consequence of lower stress upon adaptation to the farm conditions after group mixing, as discussed above for cortisol, APPs and GPx.

Amongst these protein groups, immunomodulatory proteins (group A in Fig 4) are all downregulated, with the exception of EFHD2 (swiprosin 1). Several of these proteins have been reported to be directly modulated by glucocorticoids (GC), as annexin 1 (ANXA1), that plays a role in GC-mediated downregulation of the early phase of the inflammatory response and promotes rearrangement of the actin cytoskeleton [44]. Leukocyte elastase inhibitor (SERPINB1), a regulator of neutrophil proteases, can be also under the regulation by GC and stress [45]. Stress, possibly mediated by GCs, induces the synthesis and release of HMGB1, HSP90B1 and S100A9. These proteins are DAMPs (damage associated molecular pattern), which are released by damaged cells and secreted by inflammatory cells [46]. HSP90B1 and plastin-2 (LCP1) [47–49] have been found to be directly regulated by cortisol in vitro in monocytes in proteomic studies [50].

One of the most striking findings is the conspicuous set of proteins involved in mRNA splicing (group C): Heterogeneous nuclear ribonucleoprotein A2B1 and K (HNRNPA2B1 and HNRNPK), tRNA-splicing ligase RtcB homolog (RTCB), and far upstream element-binding protein 2 (KHSRP, FUSE2). All these proteins, except HNRNPK, are downregulated at t2 and this may be a GC-mediated effect, since steroid hormones can affect the alternative splicing of several genes [51] and affect the processing of pre-mRNA [52,53]. Interestingly, splicing factors were widely identified by proteomic approaches in thymocytes from rats subjected to acute restraint stress [54]. The modulation of proteins involved in splicing as well as in transcription (histone 2B (HIST1H2BF)) or translation (elongation factor 1 alpha 1 (EEF1A)) definitively represent ongoing or upcoming genomic effects.

Another group of downregulated proteins are related to protein degradation and folding (group D): proteasome activator complex subunit 1 and 2 (PSME1/2), proteasome subunit alpha type-4 (PSMA4) and SUMO conjugating enzyme UBC9 (UBE2I), which are implicated in immunoproteasome assembly and are affected by GC and stress [55]. Downregulated chaperones include HSP90B1 [56], peptidyl-prolyl cis-trans isomerase A (PPIA) [57–60] and chaperonin containing TCP1, subunit 2 (Beta) (CCT2) [61,62]. These proteins are downregulated at t2, possibly related to the positive action of GC in proteasome activity and protein turnover [63,64].

On the other side, a set of proteins related to the cytoskeleton and cell-motility [65] are upregulated at t2 (group B). Several of them are components of the focal adhesion signalling pathway: ITGA2B, the integrin alpha chain 2b [66]; ILK, the integrin-linked kinase; PDZ and LIM domain protein 1(PDLIM1), a scaffold protein that brings other proteins to the cytoskeleton, and several actin-binding proteins such as vinculin (VCL), filamin A(FLNA), tropomyosin alpha 1 chain (TPM1) and the myosin regulatory light chains (MYL9, MYL12B). Other proteins related to the cytoskeleton are coronin (CORO1A, CORO1C), α-centractin (ACTR1A, ARP1), pleckstrin (PLECK) [67], testin (TES) [68] and WD repeat-containing protein 1 (WDR1) [69]. Calcium-binding proteins, as caldesmon (CALD1) [70] and calponin-2 (CNN2) [71] are also related to cell motility. The three fibrinogen chains (α, β, γ), a well characterized integrin ligand, have been found increased in our proteomic analysis. Interestingly, ITGA2B and integrin beta 3 (ITGB3) were found to be downregulated in PBMCs in infection by non-cytopathic bovine viral diarrhea virus (ncp BVDV) and upregulated by the cytopathic biotype (cp BVDV) in proteomic studies [66]. That would open the possibility of subclinical infections appearing in our study, which would lead to the activation of the actin cytoskeleton signalling at t2. The mechanism for the upregulation of the cytoskeletal system following adaptation to the farm may be also related to the decrease in endogenous cortisol. Indeed, Flint et al. reported, by using a proteomic approach, that stress hormones in mice provoke a decrease in actin and actin-associated proteins of the cytoskeleton in T lymphocytes, and decrease their migration ability [72].

It is worth to note that there is little overlapping between proteins identified in the present work and the proteins identified in thymocytes (which may be comparable to PBMC in the sense that PBMC contain 70% T lymphocytes) from rats subjected to short acute restraint stress. In this case, the study was designed to investigate the immediate, non-genomic effects of cortisol on the translocation of proteins between subcellular organelles, and mainly signalling proteins were identified [54]. These results indicate that most cellular changes provoked by acute exposition to cortisol differ from the exposition to variable concentrations of the hormone in a real-life, long term experiment, as the one described in the present study. Nevertheless, some main pathways regulated by cortisol are common to short as well as long-term responses to stress since several proteins were identified in both studies (HMGB1, HNRNPK, PSME1, HSP90B1 and CORO1A, and in some cases different isoforms of the same protein (PRDX2/5, PSMA1/4)). All these proteins participate in processes regulated by GCs (gene expression and splicing, immunoproteasome assembly, protein folding and danger signals).

Markers of oxidative stress are also usually altered in stress conditions [11,73,74]. In our case we have found that antioxidant peroxiredoxin-5 (PRDX5) is downregulated at t2, as well as cytosolic non-specific dipeptidase (CNDP2, also known as PepA) and cytosol aminopeptidase (LAP3) (group E). These enzymes are involved in glutathione degradation [75,76]. The differences in carbonylated proteins and SOD activity confirm the alterations in the oxidative status of PBMC throughout the experiment.

Finally, several proteins related with the metabolism or transport of purine nucleotides are also modulated, as GMP synthase (GMPS), which is upregulated at t2, whereas histidine triad nucleotide-binding protein 1 (HINT1), that hydrolyses purine nucleotides (AMP, GMP), is downregulated. That would lead to an increase in ATP concentration, which is necessary for actin polymerization and cytoskeleton dynamics. Another regulated protein involved in the transport of ATP or ADP across the mitochondrial membrane and consequently in the regulation of its availability in the cytoplasm is the small calcium binding mitochondrial carrier (SLC25A24) [77]. Two enzymes from the Krebs cycle, malate dehydrogenase (MDH1) and aconitate hydratase (ACO2) are downregulated, whereas monoglyceride lipase (MGLL) is upregulated, suggesting an adaptation of energy metabolism (group F).

**Fig 8:**
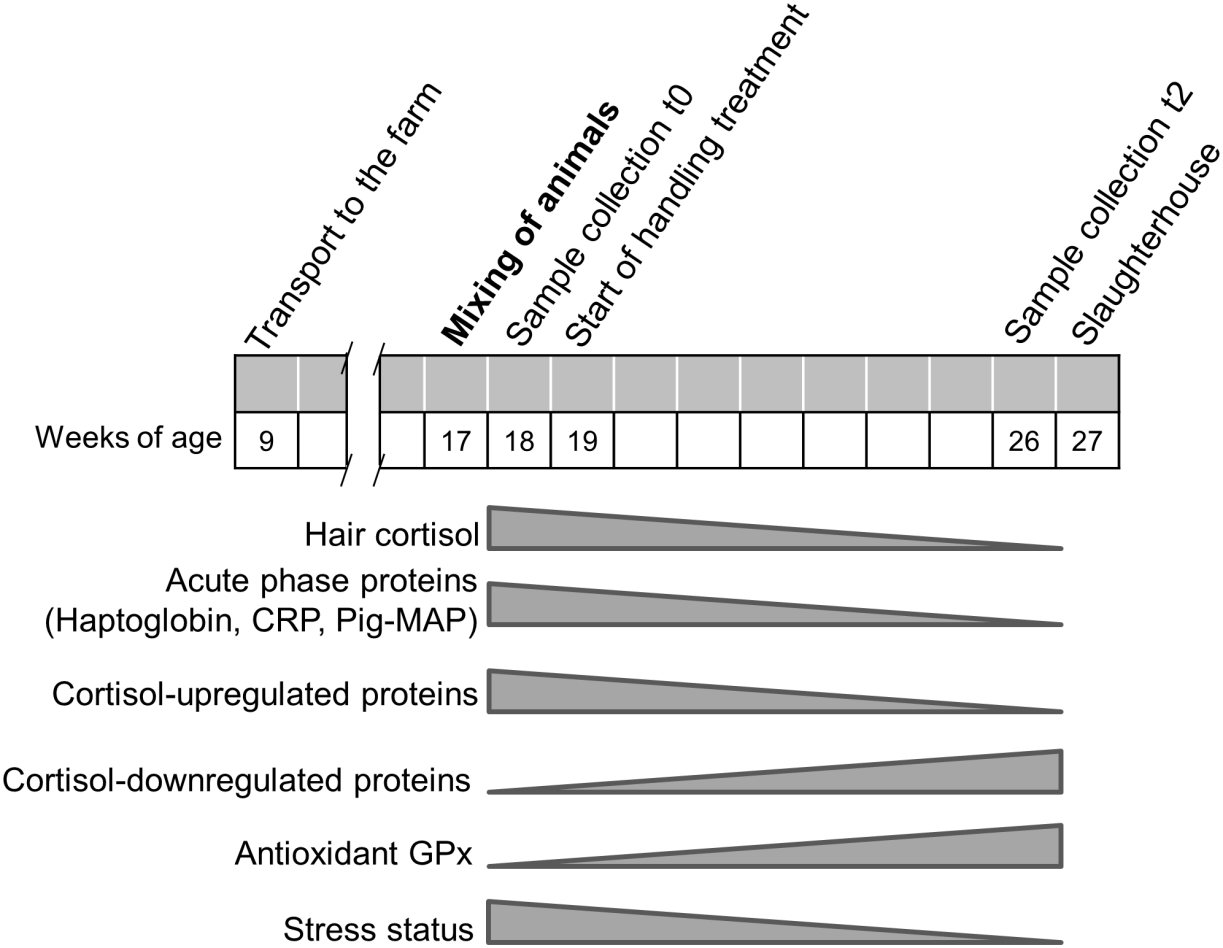
Diagram showing the conclusions of the present work. Changes in hair cortisol, serum acute phase proteins and GPx, and results of the proteomic analysis throughout the experimental 2-month period indicate that changes in PBMC proteins mainly reflect variations in the stress status of the pigs.

Fig 8 illustrates the conclusion of the present work. The variation in hair cortisol and serum APPs as well as the careful analysis of the identified proteins suggest that changes in protein composition in PBMC between t0 and t2 is mainly due to a decrease in the stress status of the individuals, following accommodation to the farm and the new group. According to this, our results show that a set of GC-induced proteins (groups “A”, “C” and “D”) are downregulated at t2, concomitantly to the lower concentration of hair cortisol. The upregulation at t2 of proteins related to the cytoskeleton (group “B”) may indicate that, after the stress is over, PBMC remain in a state of "readiness" so that they can potentially successfully combat injurious agents, as suggested by Frank et al. [78]. Proteome changes can be associated to shifts in the PBMC subpopulations, well described in situations of stress [79], but the aim of this study was to identify variations in a whole PBMC sample without taking into account changes in the cell type composition, in order to find new potential biomarkers on a PBMC sample. Physiological growth and/or sexual development may also contribute to the proteome changes, but those should be small in a 2-month period at the prepuberal age as indicated by the existing literature [80,81].

Regarding management, there are no clear effects of handling on the pig’s physiology, although some of our results may suggest that human care modulate the response to the environment. In the proteomic analysis, the NH group shows more differences than the PH group in number of differentially expressed proteins, and quantitative and qualitative (GO) variations. Animals raised in PH show a lower oxidative stress indicated by higher SOD ratio between t2 and t0. In particular, serotonin (5-HT) increases in the PFC and decreases in the amygdala in animals with PH. It is well-known that the serotonergic system is involved in the regulation of mood, stress, aggressive behaviour and mental disorders in humans and other species [34,82]. In pigs, a decrease in 5-HT has been associated to negative states as stress, fear and aggression [33,83–86] whereas an increase in 5-HT in the PFC has been related to positive conditions in the rat [87,88]. Controversial results have been reported for the amygdala [89,90]. The present study has been performed in parallel to another study in these same pigs regarding to their performance in behavioural tests (the cognitive bias test (CBT), the novel object test (NOT) and the defence cascade test (DCT)) and it did not show either an effect of handling in the tests responses. It is plausible that the handling treatment applied in the present study was not sufficiently intense or long to clearly alter the pigs’ physiological response. Lack of effect on cortisol, APP and GPx concentration due to handling would support this possibility.

In conclusion, our results show that many of the identified proteins in the proteomic approach are targets of GCs and, hence, indicate that changes in the PBMC proteome mirror the variations of endogenous cortisol and the degree of stress, since they vary concomitantly with hair cortisol and APPs (Fig 8). Taken together, these findings suggest that changes in the PBMC proteome may be sensitive indicators of animal stress. While still preliminary, the results described in the present work indicate a complex relationship between stress, handling, immune cells and brain neurotransmitters and indicate that further research in these topics should be worthwhile.

## Supporting information

S1 Table List of identified proteins after DIGE labelling of porcine PBMC. (XLSX)

S2 Table Peptide list of the identified PBMC proteins. (XLSX)

S3 Table Brain neurotransmitters concentration. (XLSX)
